# Supplementary material for: Evaluating flow modulating treatment response in intracranial aneurysms using black-blood MRI in vitro
Source: Commun Med (Lond). 2026 Mar 27;6:170. doi: 10.1038/s43856-026-01413-z (PMC13031954; doi:10.1038/s43856-026-01413-z)
Supplement: Supplementary file 2 — Supplementary Information [file 43856_2026_1413_MOESM2_ESM.pdf]

Supplementary Material for the manuscript entitled "Evaluating flow modulating treatment response in intracranial aneurysms using black-blood MRI in vitro."

PhD Mariya S. Pravdivtseva[1], Hivnu Toraman[2], PhD Jana Korte[3,4], PhD Franziska Gaidzik[3,4], B. Eng. Oluwabusayo A. Oni[5] , PhD Philipp Berg[3,6], PhD Prasanth Velvaluri[7], M. Eng. Lana Bautz[1], M. D. Fritz Wodarg[2], PhD Jan-Bernd Hövener[1,8], M. D. Olav Jansen[2], M. D. Naomi Larsen[2]

[1]Department of Radiology and Neuroradiology, Section Biomedical Imaging, University Hospital Schleswig-Holstein (UKSH), Kiel University, Kiel, Germany

[2]Department of Radiology and Neuroradiology, UKSH, Kiel University, Kiel, Germany

[3]Research Campus STIMULATE, University of Magdeburg, Magdeburg, Germany

[4]Department of Fluid Dynamics and Technical Flows, University of Magdeburg, Magdeburg, Germany

[5]Carle Illinois College of Medicine, University of Illinois Urbana-Champaign, Urbana, Illinois, United States of America

[6]Department of Medical Engineering, University of Magdeburg, Magdeburg, Germany

[7]Chair of Inorganic Functional Materials, Kiel University, Kiel, Germany

[8]Molecular Imaging North Competence Center (MOIN CC), Kiel, Germany

Corresponding author

Mariya S. Pravdivtseva

Email: mariya.pravdivtseva@rad.uni-kiel.de

Phone: +49 431 500 16 533

Fax: +49 431 500 16504

Address: Arnold-Heller-Straße 3, Haus 41, D-24105 Kiel, Germany

1. Supplementary “Materials and Methods”

1.1. In vitro vascular models

Small markers were integrated into the models and designed to slightly protrude from the vessel wall (≈ 1–2 mm). The 3D-printing material itself does not produce a visible magnetic resonance imaging (MRI) signal and therefore appears as a signal void on MR images; in combination with the surrounding agarose gel, the markers created sufficient contrast to enable reproducible MRI examination planning. Flow connectors were incorporated to integrate the models into a flow loop (Fusion 360 2.0, Autodesk), as illustrated in Supplementary Figure 1. More details were published elsewhere[1–3].

1.2. Experimental flow setup

The vascular models were connected to a closed-loop flow system, which consisted of a fluid reservoir, pump, pressure and flow sensors, and tubing. The pump and fluid reservoirs were located in the operator room. During the MRI experiment, vascular models were placed at the isocenter of the MRI system and connected to the pumps through 3-meter-long reinforced tubing with an inner diameter of 6 mm, routed through the wall access port. Near the model inlet and outlets, the reinforced tubing was transitioned to silicone tubing to facilitate sensor placement and flexibility (Supplementary Figure 2a). During pressure and flow measurements using the pressure and flow sensors, the vascular models were located in the operator room (Supplementary Figure 2a). The representative flow and pressure profiles are shown in Supplementary Figures 3 and 4, respectively.

2. Supplementary “Results”

- Numerical results of black-blood (BB) signal measurements ($BB_{\perp}$, $BB_{\perp}\ MSDE$, $BB_{\parallel}$, and $BB_{\parallel}\ MSDE$) and velocity obtained with 2D phase-contrast (PC) MRI in straight vessel models with flow rates from 0 to 5.4 ml/s are provided in the Supplementary Table 1.
- Numerical results of BB signal measurements ($BB_{\perp}$, $BB_{\perp}\ MSDE$, $BB_{\parallel}$, and $BB_{\parallel}\ MSDE$) and velocity obtained with 4D flow MRI in the internal carotid artery (ICA) model with

flow-diverter stent (FD1) and without any device supplied with flow rates of 4.5 ml/s are provided in the Supplementary Table 2.

- Numerical results of BB signal measurements ($BB_{\perp}$, $BB_{\perp}\ MSDE$, $BB_{\parallel}$, and $BB_{\parallel}\ MSDE$) in the ICA model with FD1 and without any device supplied, with no flow, are provided in the Supplementary Table 3 and visualized in Supplementary Figure 5.
- Numerical results of BB signal measurements ($BB_{\perp}$, $BB_{\perp}\ MSDE$) and velocity obtained with 4D flow MRI in the ICA model supplied with a flow rate of 4.5 ml/s with FD1-5 and without any device within the aneurysm and vessel volumes of interest (VOIs) are provided in the Supplementary Table 4. Statistical analysis of these results is provided in the Supplementary Table 5. The BB signal distribution within the vessel VOI is visualized with violin plots in Supplementary Figure 6.
- Numerical results of BB signal measurements ($BB_{\perp}$) and velocity obtained with 4D flow MRI in basilar artery (BA) models supplied with a flow rate of 2.4 ml/s with intrasaccular flow-disrupting devices (IFD1-10) and without any device within the aneurysm and vessel VOI are provided in the Supplementary Table 6. Statistical analysis of these results is provided in the Supplementary Table 7. The BB signal distribution within the vessel VOI is visualized with violin plots in Supplementary Figure 7.
- Statistical analysis of treatment effects—relative velocity changes and BB signal variations induced by FD1–5 and IFD1–10 compared with the control—is summarized in Supplementary Table 8.

3. Supplementary "Figures"

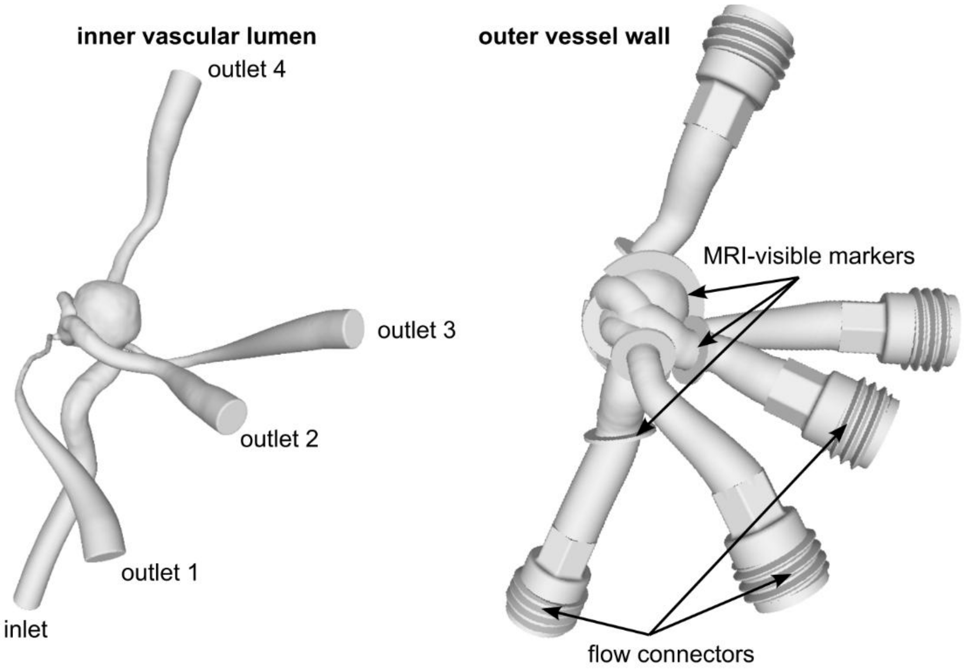

Supplementary Figure 1. *3D rendering of the internal carotid artery (ICA) model showing the inner vascular lumen (left) and the outer vessel wall with additional structures used for 3D printing and later in magnetic resonance imaging (MRI) experiments (right). The ICA model features one inlet and four outlets. The outer wall includes integrated flow connectors to facilitate connection to the experimental flow setup and MRI-visible markers to enable reproducible MRI planning and postprocessing.*

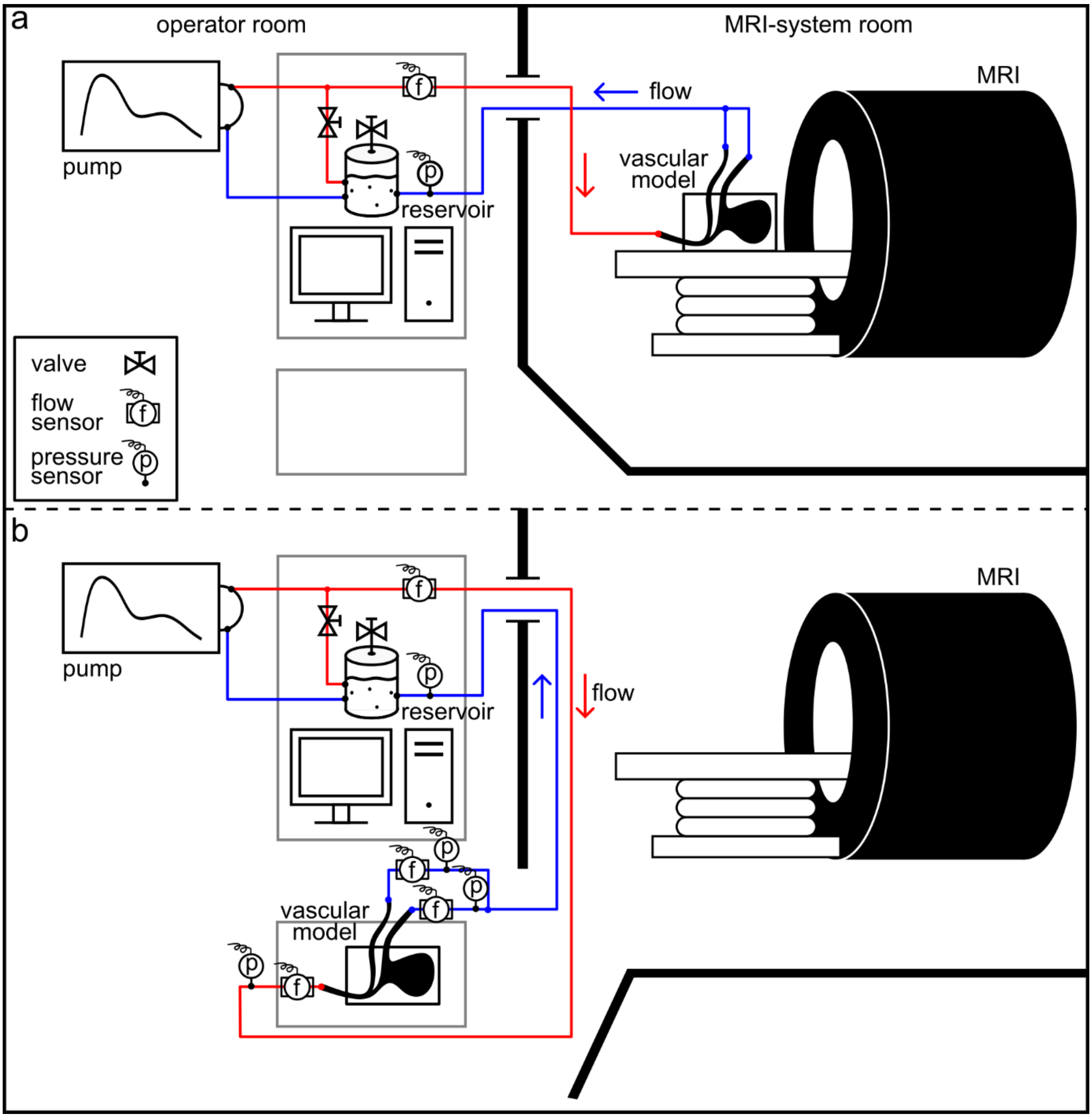

Supplementary Figure 2. *Schematic illustration of the experimental flow setup during MRI acquisition (a) and during flow and pressure measurements (b). In (a), the pump and fluid reservoir were located in the operator room, while the vascular models were positioned at the MRI isocenter. In (b), pressure and flow were measured at the inlet and outlets of the vascular model, also in the operator room. Flow rates were adjusted by varying the pump speed and the bypass resistance using a valve. Although pressure was not actively controlled, any pressure buildup was passively released via the valve connected to the water reservoir. Red lines indicate the flow path from the pump to the reservoir and the vascular model, while blue lines indicate the return flow from the vascular model to the reservoir and from the reservoir back to the pump.*

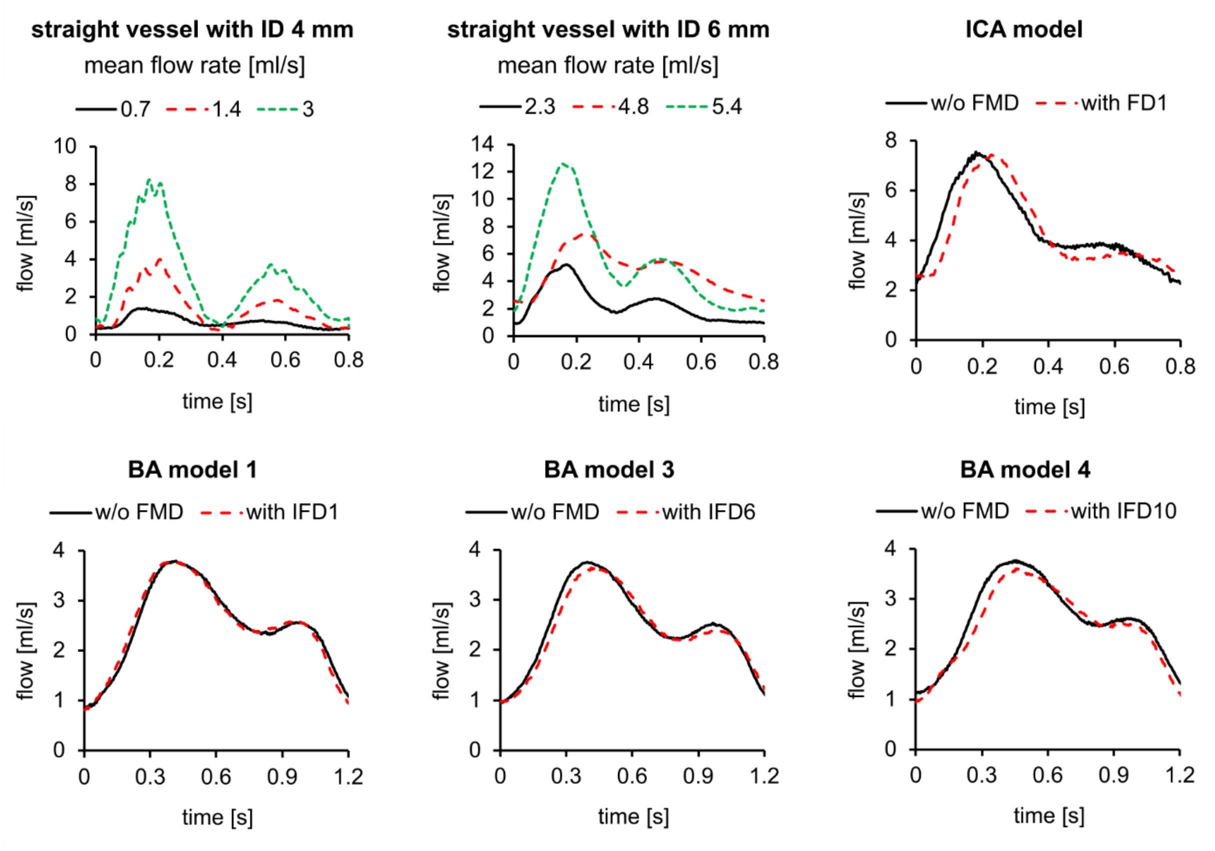

Supplementary Figure 3. *Representative flow profiles acquired at the inlets of the straight vessel model, which was supplied with mean flow rates of 0.7, 1.4, and 3.0 ml/s for an internal diameter (ID) of 4 mm, and 2.3, 4.8, and 5.4 ml/s for an ID of 6 mm. Flow profiles are also shown for intracranial aneurysm models, both with and without (w/o) implanted flow-modulation devices (FMDs). The internal carotid artery (ICA) models were supplied with a mean flow rate of 4.5 ml/s, and the basilar artery (BA) models with 2.4 ml/s, mimicking physiologically relevant inlet conditions.*

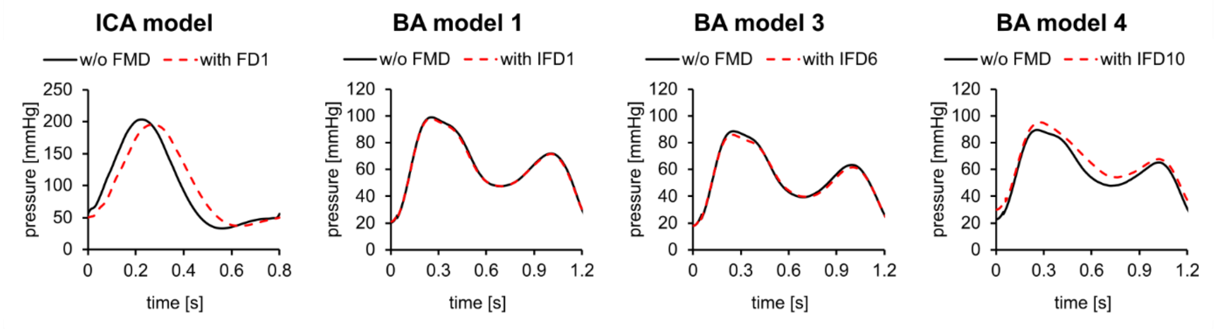

Supplementary Figure 4. *Representative pressure profiles acquired at the inlets of the intracranial aneurysm models, both with and without (w/o) implanted flow-modulation devices (FMDs). The internal carotid artery (ICA) models were supplied with a mean flow rate of 4.5 ml/s, and the basilar artery (BA) models with 2.4 ml/s, mimicking physiologically relevant inlet conditions.*

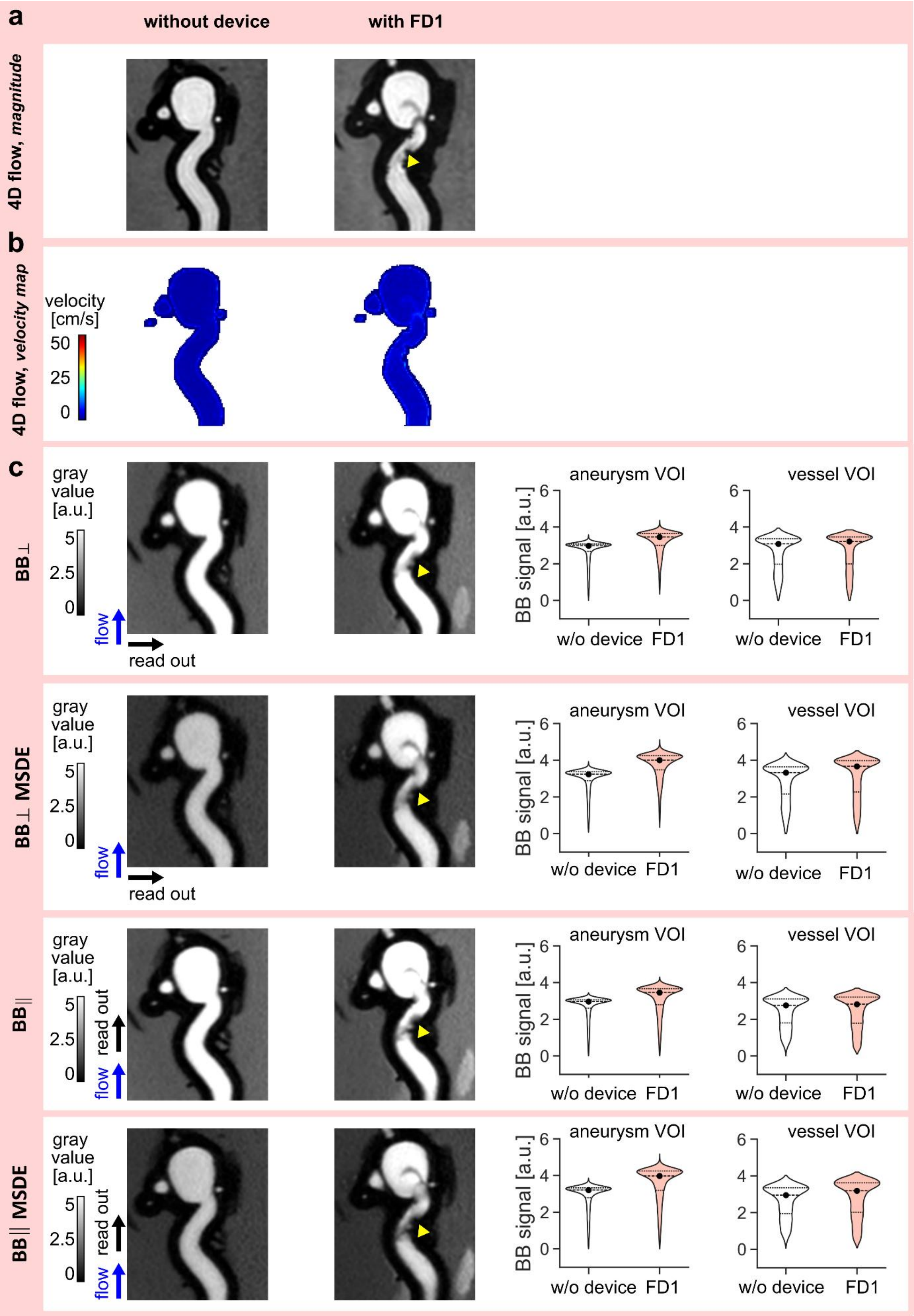

Supplementary Figure 5. *Effect of the presence of a flow-diverter stent (FD1) on velocity and black-blood (BB) signal in an internal carotid artery (ICA) model supplied with no flow. Representative images include 4D flow*

magnitude MRI (a), velocity maps (b), and BB MRI sequences (c). FD1 caused a minor metal artifact at the parent vessel (yellow arrowhead). No changes in BB signal and velocity were observed regardless of the presence of FD1.

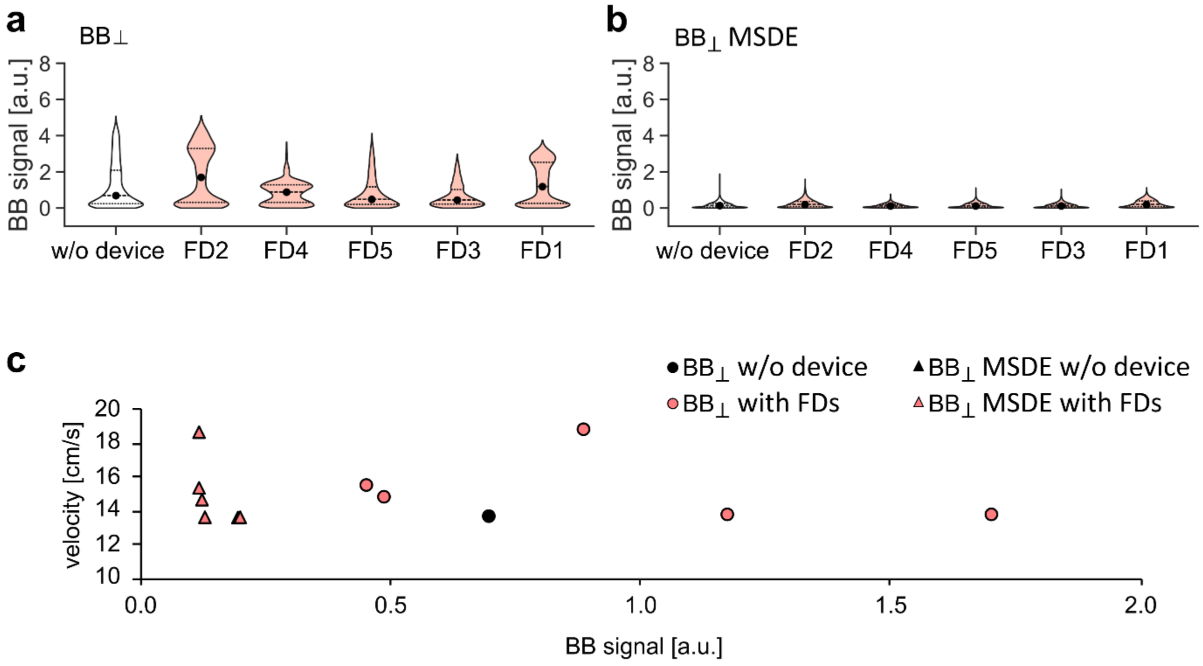

Supplementary Figure 6. *Effect of flow-diverter stents (FD1-5) on velocity and black-blood (BB) signal in the parent vessel of the internal carotid artery (ICA) model. Violin plots illustrate the BB signal in the parent vessel, depicted by* $BB_{\perp}$ *(a) and* $BB_{\perp}\ MSDE$ *(b) with FD1-5 and without FD. A scatter plot illustrates the relationship between the median value of spatially averaged velocity and the median value of the BB signal in the parent vessel (c).* $BB_{\perp}\ MSDE$ *resulted in a lower BB signal in comparison* $BB_{\perp}$*. No apparent effect of FDs on the BB signal was observed, although all resulting distributions of BB signal within a parental vessel were statistically different. Statistical analysis was performed using the Kruskal–Wallis test, followed by post hoc Dunn pairwise comparisons with Benjamini–Hochberg p-value adjustment.*

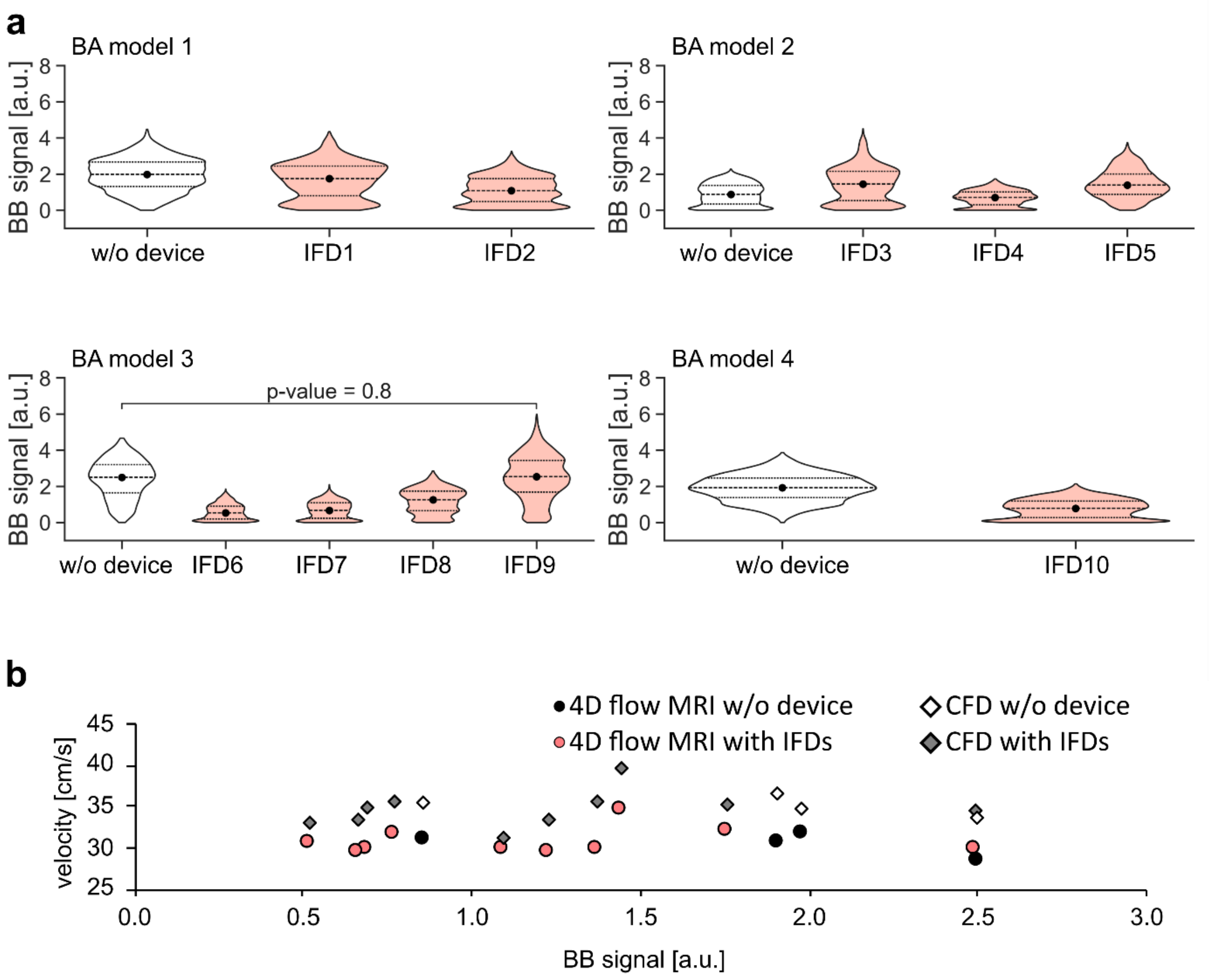

Supplementary Figure 7. *Effect of intrasaccular flow-disrupting devices (IFD1-10) on velocity and black-blood (BB) signal in the parent vessel of the basilar artery (BA) models. Violin plots illustrate the BB signal in the parent vessel, depicted by* $BB_{\perp}$ *(a) with IFD1-10 and without IFD. A scatter plot illustrates the relationship between the median value of spatially averaged velocity and the median value of the BB signal in the parent vessel (b). No clear effect of FDs on the BB signal in the parent vessel was observed. Only for the BA model 3, the pair of without (w/o) IFD and with IFD9 resulted in statistically similar distribution of BB signal within parental vessel (p-value = 0.8), while the others were different (p-value < 0.05). Statistical analysis was performed using the Kruskal–Wallis test, followed by post hoc Dunn pairwise comparisons with Benjamini–Hochberg p-value adjustment.*

4. Supplementary “Tables”

Supplementary Table 1. *Cumulative statistics for the black-blood (BB) signal in the straight vessels supplied with varied flow rates.*

vessel ID	pump flow [ml/s]	2D PC MRI, velocity [cm/s]			$BB_{\perp}$ [a.u.]			$BB_{\perp}\ MSDE$ [a.u.]			$BB_{\parallel}$ [a.u.]			$BB_{\parallel}\ MSDE$ [a.u.]		
		**Median**	Q1	Q3	**Median**	Q1	Q3	**Median**	Q1	Q3	**Median**	Q1	Q3	**Median**	Q1	Q3
4	0	**0.0**	0.0	0.0	**2.83**	2.10	3.06	**3.04**	2.27	3.32	**2.49**	1.88	2.79	**2.67**	2.03	3.02
	0.7	**5.4**	3.2	7.6	**2.90**	2.13	3.12	**0.25**	0.15	0.50	**0.11**	0.05	0.17	**0.06**	0.02	0.13
	1.4	**12.3**	6.9	16.3	**2.13**	1.60	2.53	**0.21**	0.13	0.29	**0.04**	0.01	0.07	**0.06**	0.03	0.12
	3	**25.5**	15.0	35.4	**1.16**	0.84	1.57	**0.09**	0.03	0.16	**0.03**	0.01	0.06	**0.05**	0.02	0.10
	4.7	**36.5**	19.8	52.5	**0.73**	0.35	1.13	**0.08**	0.03	0.14	**0.03**	0.01	0.07	**0.04**	0.02	0.10
6	0	**0.0**	0.0	0.0	**2.74**	1.80	2.93	**2.74**	1.82	2.96	**2.60**	2.09	2.80	**2.58**	2.10	2.80
	1	**4.1**	2.9	5.0	**2.82**	1.86	3.00	**0.26**	0.09	0.45	**0.21**	0.14	0.28	**0.14**	0.06	0.23
	2.3	**7.6**	4.4	12.3	**2.72**	1.86	2.88	**0.30**	0.17	0.47	**0.11**	0.07	0.15	**0.12**	0.07	0.17
	4.7	**17.9**	12.6	23.6	**2.47**	1.77	2.75	**0.24**	0.16	0.32	**0.06**	0.03	0.10	**0.07**	0.04	0.11
	5.4	**18.0**	9.8	31.3	**2.17**	1.62	2.48	**0.21**	0.14	0.27	**0.07**	0.03	0.10	**0.05**	0.02	0.10
Abbreviations: Q1 and Q3 – first and third quantiles, ID – inner diameter, BB – black-blood, PC – phase-contrast, MSDE – motion-sensitized driven-equilibrium, a.u. – arbitrary units																

Supplementary Table 2. *Cumulative statistics for velocity measured with 4D flow magnetic resonance imaging (MRI) and black-blood (BB) signal in the internal carotid artery (ICA) model with flow-diverter stent 1 (FD1) and without FD supplied with a mean flow rate of 4.5 ml/s. A Kruskal-Wallis, followed by post hoc Dunn pairwise comparisons with Benjamini–Hochberg p-value adjustment test was performed to assess voxel-wise differences in BB signal within the aneurysm or parent vessel, with and without the device.*

VOI	device	4D flow MRI, velocity [cm/s]			$BB_{\perp}$ [a.u.]			$BB_{\perp}$ $MSDE$ [a.u.]			$BB_{\parallel}$ [a.u.]			$BB_{\parallel}$ $MSDE$ [a.u.]		
		**Median**	Q1	Q3	**Median**	Q1	Q3	**Median**	Q1	Q3	**Median**	Q1	Q3	**Median**	Q1	Q3
aneurysm	w/o FD	**11.47**	9.76	15.13	**0.15**	0.08	0.28	**0.07**	0.03	0.13	**0.09**	0.04	0.15	**0.06**	0.03	0.12
	FD1	**0.67**	0.67	0.69	**3.41**	2.92	3.60	**3.88**	3.29	4.11	**3.35**	2.66	3.56	**3.74**	2.93	4.02
	statistics				38411.01			38467.88			39632.18			39893.64		
	p-value				3.56E-128			0			0			0		
vessel	w/o FD	**14.75**	12.93	20.87	**0.53**	0.23	1.03	**0.11**	0.04	0.19	**0.05**	0.02	0.10	**0.08**	0.03	0.14
	FD1	**15.27**	14.39	23.05	**0.43**	0.18	1.02	**0.10**	0.03	0.18	**0.05**	0.02	0.10	**0.07**	0.02	0.13
	statistics				102.89			202.16			6.94792			19.61822		
	p-value				3.54E-24			7.05E-46			8.39E-03			9.46E-06		

Abbreviations: FD – flow-diverter stent, ICA – internal carotid artery, Q1 and Q3 – first and third quantiles, ID – inner diameter, BB – black-blood, MSDE – motion-sensitized driven-equilibrium, a.u. – arbitrary units, w/o – without, MRI – magnetic resonance imaging

Supplementary Table 3. *Cumulative statistics for the black-blood (BB) signal in the internal carotid artery (ICA) model with flow-diverter stent (FD1) and without FD supplied with no flow. A Kruskal-Wallis, followed by post hoc Dunn pairwise comparisons with Benjamini–Hochberg p-value adjustment test was performed to assess voxel-wise differences in BB signal within the aneurysm or parent vessel, with and without the device.*

VOI	device	$BB_{\perp}$ [a.u.]			$BB_{\perp}\ MSDE$ [a.u.]			$BB_{\parallel}$ [a.u.]			$BB_{\parallel}\ MSDE$ [a.u.]		
		**Median**	Q1	Q3	**Median**	Q1	Q3	**Median**	Q1	Q3	**Median**	Q1	Q3
aneurysm	w/o FD	**2.99**	2.68	3.08	**3.25**	2.89	3.37	**2.99**	2.62	3.08	**3.21**	2.80	3.33
	FD1	**3.46**	3.01	3.65	**4.03**	3.50	4.27	**3.46**	2.79	3.67	**3.97**	3.18	4.23
	stats.	12882.25			16617.27			9704.38			12524.96		
	p-val.	0			0			0			0		
vessel	w/o FD	**3.10**	1.98	3.37	**3.33**	2.15	3.64	**2.78**	1.80	3.13	**2.97**	1.96	3.36
	FD1	**3.22**	1.99	3.46	**3.69**	2.29	4.00	**2.82**	1.78	3.21	**3.18**	2.02	3.61
	stats.	168.3			1273.81			74.82			639.63		
	p-val.	1.74E-38			5.55E-279			5.15E-18			4.02E-141		
Abbreviations: FD – flow-diverter stent, ICA – internal carotid artery, Q1 and Q3 – first and third quantiles, ID – inner diameter, BB – black-blood, MSDE – motion-sensitized driven-equilibrium, a.u.- arbitrary units, w/o – without.													

Supplementary Table 4. *Cumulative statistics for velocity measured with 4D flow magnetic resonance imaging (MRI) and black-blood (BB) signal in the internal carotid artery (ICA) model with flow-diverter stents (FD1-5) supplied with a mean flow rate of 4.5 ml/s. The results for FD1-5 are arranged from the worst aneurysmal flow reduction to the best.*

VOI	device	4D flow MRI, velocity [cm/s]			$BB_{\perp}$ [a.u.]			$BB_{\perp}\ MSDE$ [a.u.]		
		**Median**	Q1	Q3	**Median**	Q1	Q3	**Median**	Q1	Q3
aneurysm	w/o FD	**12.2**	10.9	18.5	**0.10**	0.04	0.18	**0.09**	0.05	0.17
	FD2	**11.2**	9.4	15.0	**0.08**	0.03	0.16	**0.08**	0.04	0.17
	FD4	**5.0**	4.3	5.3	**0.26**	0.13	0.50	**0.14**	0.06	0.29
	FD5	**3.5**	3.0	4.0	**0.48**	0.25	0.84	**0.25**	0.10	0.56
	FD3	**1.9**	1.6	2.5	**1.97**	1.12	2.96	**0.93**	0.39	1.72
	FD1	**0.6**	0.6	0.6	**4.12**	2.77	4.35	**4.68**	3.10	4.99
vessel	w/o FD	**13.6**	11.7	22.4	**0.70**	0.23	2.09	**0.13**	0.05	0.25
	FD2	**13.5**	12.3	20.3	**1.71**	0.31	3.29	**0.19**	0.08	0.36
	FD4	**18.6**	14.1	21.0	**0.89**	0.32	1.29	**0.12**	0.05	0.21
	FD5	**14.6**	13.0	21.7	**0.49**	0.20	1.17	**0.13**	0.04	0.24
	FD3	**15.3**	13.7	22.7	**0.46**	0.22	1.02	**0.12**	0.05	0.22
	FD1	**13.6**	12.0	25.3	**1.18**	0.26	2.53	**0.20**	0.09	0.38

Abbreviations: MRI – magnetic resonance imaging, FD – flow-diverter stent, ICA – internal carotid artery, Q1 and Q3 – first and third quantiles, BB – black-blood, MSDE – motion-sensitized driven-equilibrium, VOI – volume of interest, w/o – without, a.u. – arbitrary units.

Supplementary Table 5. *P-values from Kruskal-Wallis, followed by post hoc Dunn pairwise comparisons with Benjamini–Hochberg p-value adjustment tests comparing voxel-wise black-blood (BB) signal intensity in the internal carotid artery (ICA) model with flow-diverter stents (FD1-5) supplied with a mean flow rate of 4.5 ml/s.*

MRI sequence		$BB_{\perp}$		$BB_{\perp}\ MSDE$	
VOI		aneurysm	vessel	aneurysm	vessel
**Kruskal-Wallis test**					
Statistics		141558.43	6230.22	122198.06	5090.87
p-value		0	0	0	0
**Post hoc Dunn group comparison**					
group 1	group 2	p-value	p-value	p-value	p-value
FD2	FD1	0	7.60E-104	0	1.81E-09
FD2	FD3	0	0	0	0
FD1	FD3	0	0	0	0
FD2	FD4	0	0	0	0
FD1	FD4	0	5.16E-104	0	0
FD3	FD4	0	2.10E-131	0	1.40E-02
FD2	FD5	0	0	0	5.58E-275
FD1	FD5	0	0	0	0
FD3	FD5	0	1.10E-16	0	3.50E-11
FD4	FD5	0	1.97E-55	0	9.81E-19
FD2	w/o FD	4.66E-36	6.53E-245	8.10E-12	9.83E-214
FD1	w/o FD	0	4.22E-39	0	0
FD3	w/o FD	0	2.19E-235	0	4.29E-27
FD4	w/o FD	0	2.99E-16	0	5.89E-38
FD5	w/o FD	0	1.46E-125	0	5.21E-05
Abbreviations: FD – flow-diverter stent, BB – black-blood, MSDE – motion-sensitized driven-equilibrium, VOI – volume of interest, w/o – without.					

Supplementary Table 6. *Cumulative statistics for velocity measured with 4D flow magnetic resonance imaging (MRI) and calculated with computational fluid dynamics (CFD) and black-blood (BB) signal in the basilar artery (BA) model with intrasaccular flow-disrupting devices (IFD1-10) supplied with a mean flow rate of 2.4 ml/s.*

VOI	aneurysm model	device	device size [mm]	4D flow MRI, velocity [cm/s]			CFD, velocity [cm/s]			$BB_{\perp}$ [a.u.]		
				**Median**	Q1	Q3	**Median**	Q1	Q3	**Median**	Q1	Q3
aneurysm	BA model 1	w/o	5	**6.5**	6.1	6.8	**11.8**	8.3	15.6	**0.11**	0.06	0.18
		IFD1	5	**no data available**			**1.2**	0.6	2.2	**1.24**	0.49	2.60
		IFD2	5	**no data available**			**0.0**	0.0	0.1	**1.32**	0.53	2.60
	BA model 2	w/o	5	**2.7**	2.5	2.9	**6.2**	4.8	6.8	**0.58**	0.27	1.04
		IFD3	5	**2.3**	2.1	2.3	**0.2**	0.2	0.3	**2.81**	0.79	4.52
		IFD4	5	**1.8**	1.7	1.9	**0.5**	0.3	0.7	**0.77**	0.17	1.86
		IFD5	5	**2.0**	2.0	2.2	**0.3**	0.2	0.4	**2.01**	0.60	3.63
	BA model 3	w/o	11	**16.4**	14.7	19.3	**18.4**	14.6	25.0	**0.05**	0.02	0.11
		IFD6	11	**1.9**	1.9	1.9	**6.7**	5.2	9.8	**3.61**	2.56	4.49
		IFD7	11	**2.0**	2.0	2.0	**4.1**	3.3	5.7	**4.13**	2.84	4.79
		IFD8	11	**1.9**	1.8	1.9	**5.2**	4.1	7.0	**4.05**	3.07	4.46
		IFD9	11	**1.9**	1.9	2.0	**5.0**	3.9	6.1	**5.62**	2.89	6.06
	BA model 4	w/o	14	**10.2**	8.3	13.1	**14.3**	11.6	18.8	**0.09**	0.04	0.19
		IFD10	14	**3.7**	3.2	4.1	**3.2**	2.1	4.0	**1.38**	0.67	2.10
vessel	BA model 1	w/o	5	**31.7**	28.4	39.7	**34.8**	25.0	45.2	**1.98**	1.31	2.67
		IFD1	5	**31.6**	28.0	39.5	**35.0**	25.2	46.5	**1.76**	0.80	2.44
		IFD2	5	**29.5**	28.3	36.4	**30.9**	25.0	41.6	**1.09**	0.47	1.75
	BA model 2	w/o	5	**30.9**	28.6	38.3	**35.4**	25.7	45.5	**0.86**	0.34	1.35
		IFD3	5	**34.2**	27.1	37.4	**39.4**	29.8	43.4	**1.45**	0.52	2.14
		IFD4	5	**29.5**	27.2	36.0	**34.6**	25.1	42.5	**0.70**	0.31	1.00
		IFD5	5	**29.7**	26.8	35.4	**35.6**	25.9	45.6	**1.37**	0.87	1.98
	BA model 3	w/o	11	**28.4**	26.7	36.1	**33.7**	25.5	45.6	**2.50**	1.64	3.19
		IFD6	11	**30.3**	28.2	37.5	**32.9**	26.0	43.8	**0.53**	0.18	0.90
		IFD7	11	**29.3**	27.7	37.3	**33.2**	26.2	45.3	**0.66**	0.21	1.06
		IFD8	11	**29.2**	27.4	36.4	**33.3**	26.2	44.2	**1.23**	0.64	1.71
		IFD9	11	**29.5**	27.8	36.8	**34.3**	25.6	44.6	**2.50**	1.66	3.39
	BA model 4	w/o	14	**30.8**	27.9	38.8	**36.4**	27.3	46.7	**1.91**	1.35	2.44
		IFD10	14	**31.3**	28.0	37.6	**35.6**	24.7	45.1	**0.77**	0.27	1.17

Abbreviations: IFD – intrasaccular flow-disrupting device, BA – basilar artery, Q1 and Q3 – first and third quantiles, BB – black-blood, VOI – volume of interest, w/o – without, a.u. – arbitrary units, CFD – computational fluid dynamics, MRI – magnetic resonance imaging.

Supplementary Table 7. *P-values from Kruskal-Wallis, followed by post hoc Dunn pairwise comparisons with Benjamini–Hochberg p-value adjustment tests comparing voxel-wise black-blood (BB) signal intensity in the basilar artery (BA) model with intrasaccular flow-disrupting devices (IFD1-10). Statistically similar distributions (p-value > 0.05) of BB signal within volume of interest (VOI) are highlighted in bold.*

aneurysm model	VOI		aneurysm	vessel
BA model 1	**Kruskal-Wallis test**			
	statistics		586.92	2328.99
	p-value		0	0
	**Post hoc Dunn group comparison**			
	group 1	group 2	p-value	p-value
	IFD1	IFD2	**4.91E-01**	1.14E-206
	IFD1	w/o IFD	4.32E-107	6.31E-64
	IFD2	w/o IFD	1.30E-93	0
BA model 2	**Kruskal-Wallis test**			
	Statistics		759.11228	3868.76943
	p-value		0	0
	**Post hoc Dunn group comparison**			
	group 1	group 2	p-value	p-value
	IFD3	w/o IFD	7.39E-122	5.34E-231
	IFD3	IFD4	2.30E-82	0
	w/o IFD	IFD4	4.69E-06	1.09E-59
	IFD3	IFD5	4.69E-06	2.71E-09
	w/o IFD	IFD5	2.12E-76	0
	IFD4	IFD5	3.70E-46	0
BA model 3	**Kruskal-Wallis test**			
	Statistics		15234.29	13769.83
	p-value		0	0
	**Post hoc Dunn group comparison**			
	group 1	group 2	p-value	p-value
	IFD6	IFD7	6.46E-33	3.94E-11
	IFD6	w/o IFD	0	0
	IFD7	w/o IFD	0	0
	IFD6	IFD8	4.13E-11	0
	IFD7	IFD8	8.49E-08	8.09E-210
	w/o IFD	IFD8	0	0
	IFD6	IFD9	0	0
	IFD7	IFD9	7.64E-139	0
	w/o IFD	IFD9	0	**7.78E-01**
	IFD8	IFD9	6.24E-207	0
BA model 4	**Kruskal-wallis test**			
	Statistics		21788.78102	4682.84644
	p-value		0	0
Abbreviations: IFD – intrasaccular flow-disrupting device, BA – basilar artery, BB – black-blood, VOI – volume of interest, w/o – without.				

Supplementary Table 8. *Cumulative statistics for the treatment effect of flow modulation devices on black-blood signal (ΔBB) and velocity (Δvelocity) in internal carotid artery (ICA) models without and with flow-diverter stents (FD1-5) and basilar artery (BA) models without and with intrasaccular flow-disrupting devices (IFD1-10).*

**#**	**data**	ΔBB		Δvelocity	
	**VOI**	**aneurysm**	**vessel**	**aneurysm**	**vessel**
**1**	**FD1**	21.03	-0.18	-0.94	0.04
**2**	**FD1**	41.33	0.69	-0.95	0.00
**3**	**FD2**	-0.14	1.44	-0.08	-0.01
**4**	**FD3**	19.22	-0.35	-0.84	0.12
**5**	**FD4**	1.68	0.27	-0.59	0.37
**6**	**FD5**	3.95	-0.30	-0.71	0.07
**7**	**IFD1**	10.27	-0.11	-	0.00
**8**	**IFD2**	11.03	-0.45	-	-0.07
**9**	**IFD3**	3.84	0.69	-0.16	0.11
**10**	**IFD4**	0.33	-0.18	-0.34	-0.05
**11**	**IFD5**	2.46	0.60	-0.25	-0.04
**12**	**IFD6**	67.52	-0.79	-0.88	0.07
**13**	**IFD7**	77.47	-0.74	-0.88	0.03
**14**	**IFD8**	75.92	-0.51	-0.89	0.03
**15**	**IFD9**	105.84	0.00	-0.88	0.04
**16**	**IFD10**	14.63	-0.60	-0.64	0.02
**Median**		16.92	-0.18	-0.78	0.03
**Q1**		3.50	-0.46	-0.88	0.00
**Q3**		47.87	0.36	-0.40	0.07
Abbreviations: FD – flow-diverter stent, IFD – intrasaccular flow-disrupting device, Q1 and Q3 – first and third quantiles, BB – black-blood, VOI – volume of interest.					

5. Supplementary "References"

1. Pravdivtseva, M. S. *et al.* 3D-printed, patient-specific intracranial aneurysm models: From clinical data to flow experiments with endovascular devices. *Med Phys* **48**, 1469–1484 (2021).
2. Pravdivtseva, M. S. *et al.* The effect of the size of the new contour neurovascular device for altering intraaneurysmal flow. *Interv Neuroradiol* 15910199221145985 (2023) doi:10.1177/15910199221145985.
3. Velvaluri, P. *et al.* Thin-Film Patient-Specific Flow Diverter Stents for the Treatment of Intracranial Aneurysms. *Adv Mater Technol* **6**, 2100384 (2021).
