# Supplementary material for: Evaluating flow modulating treatment response in intracranial aneurysms using black-blood MRI in vitro
Source: Commun Med (Lond). 2026 Mar 27;6:170. doi: 10.1038/s43856-026-01413-z (PMC13031954; doi:10.1038/s43856-026-01413-z)
Supplement: Supplementary file 3 — Description of Additional Supplementary files [file 43856_2026_1413_MOESM3_ESM.pdf]

Description of Additional Supplementary Files

File name: Supplementary Data 1-11

Description: The processed data underlying the plots in the figures
